# Attention-Based UNet Deep Learning Model for Plaque Segmentation in Carotid Ultrasound for Stroke Risk Stratification: An Artificial Intelligence Paradigm

**DOI:** 10.3390/jcdd9100326

**Published:** 2022-09-27

**Authors:** Pankaj K. Jain, Abhishek Dubey, Luca Saba, Narender N. Khanna, John R. Laird, Andrew Nicolaides, Mostafa M. Fouda, Jasjit S. Suri, Neeraj Sharma

**Affiliations:** 1 School of Biomedical Engineering, Indian Institute of Technology (BHU), Varanasi 221005, India; 2 Department of Electronics and Communication, Shree Mata Vaishno Devi University, Jammu 182301, India; 3 Department of Radiology, Azienda Ospedaliero Universitaria (A.O.U.), 09100 Cagliari, Italy; 4 Department of Cardiology, Indraprastha APOLLO Hospital, New Delhi 110076, India; 5 Heart and Vascular Institute, Adventist Heath St. Helena, St. Helena, CA 94574, USA; 6 Vascular Screening and Diagnostic Centre and University of Nicosia Medical School, Nicosia 2409, Cyprus; 7 Department of Electrical and Computer Engineering, Idaho State University, Pocatello, ID 83209, USA; 8 Stroke Diagnostic and Monitoring Division, AtheroPoint™, Roseville, CA 95661, USA

**Keywords:** atherosclerosis, stroke, CVD, ICA, CCA, plaque segmentation, deep learning, UNet, UNet++, UNet+++, Attention-UNet

## Abstract

Stroke and cardiovascular diseases (CVD) significantly affect the world population. The early detection of such events may prevent the burden of death and costly surgery. Conventional methods are neither automated nor clinically accurate. Artificial Intelligence-based methods of automatically detecting and predicting the severity of CVD and stroke in their early stages are of prime importance. This study proposes an attention-channel-based UNet deep learning (DL) model that identifies the carotid plaques in the internal carotid artery (ICA) and common carotid artery (CCA) images. Our experiments consist of 970 ICA images from the UK, 379 CCA images from diabetic Japanese patients, and 300 CCA images from post-menopausal women from Hong Kong. We combined both CCA images to form an integrated database of 679 images. A rotation transformation technique was applied to 679 CCA images, doubling the database for the experiments. The cross-validation K5 (80% training: 20% testing) protocol was applied for accuracy determination. The results of the Attention-UNet model are benchmarked against UNet, UNet++, and UNet3P models. Visual plaque segmentation showed improvement in the Attention-UNet results compared to the other three models. The correlation coefficient (CC) value for Attention-UNet is 0.96, compared to 0.93, 0.96, and 0.92 for UNet, UNet++, and UNet3P models. Similarly, the AUC value for Attention-UNet is 0.97, compared to 0.964, 0.966, and 0.965 for other models. Conclusively, the Attention-UNet model is beneficial in segmenting very bright and fuzzy plaque images that are hard to diagnose using other methods. Further, we present a multi-ethnic, multi-center, racial bias-free study of stroke risk assessment.

## 1. Introduction

In recent decades, stroke and other cardiovascular diseases (CVD) have emerged as fatal diseases across the globe. For developing countries such as India, stroke has become a pandemic [1,2,3,4] in recent years with 105 to 152 cases per 0.1 million of the population [5]. In developed countries such as the USA, 795,000 people experience a stroke, and approximately 240,000 suffer a transient ischemic stroke yearly [6]. Various cardiovascular risk factors such as diabetes [7,8], smoking, hypertension, hypercholesterolemia, and irregular lifestyle have been discussed in many studies as leading to higher stroke and CVD events [6,9,10,11].

A major cause of stroke and CVD is atherosclerosis disease of the arteries [12,13], particularly in the intimal and media walls [14]. Different stages of plaque constitute soft plaque, such as lipid and macrophages, and hard plaque, such as calcium [15], or so-called plaque morphology, which is helpful in the diagnosis of atherosclerosis disease severity [16,17]. Three primary techniques available for carotid plaque identification are magnetic resonance imaging (MRI) [18], computed tomography (CT) [19], and ultrasound (US) imaging [20]. Based on its cost-effective, non-radiative, portable, and device-handling properties, ultrasound is more prevalent in carotid artery imaging [21,22]. Different grayscale levels that are generated by ultrasound echo segregate parts of the carotid artery, such as the lumen area, lumen intima layer, media-adventitia layer, and plaque, such as fibrin, fibrosis, and hard and soft plaque [23]. This also helps the sonographer or radiologist to mark the plaque area by delineating the lumen intima (LI) and media adventitia (MA) borders of the carotid arteries [24].

Delineating the carotid plaque using the manual technique is tedious and error-prone. Thus, researchers have proposed various automated methods of delineating the carotid plaques [25]. The computerized method for carotid plaque and intima-media thickness (IMT) delineation involves edge-based [26,27] methods including first order absolute moment (FOAM) [28] and local maxima [29,30]; anisotropic Gaussian derivative filtering [31,32] and non-maximum suppression [33]; snake-based [34,35,36] or dual-snake-based [36]; scale-space [37,38,39] (or AtheroEdge™ 1.0, 2.0, AtheroPoint LLC, CA, USA) [31]; shape-based [40,41]; and level-set [42,43,44,45,46,47,48]. A state-of-the-art automated segmentation system, AtheroEdge™ 2.0, was developed by AtheroPoint LLC, Roseville, CA, USA for cIMT measurement at bulb edge points [49]. Several methods have been published that benchmark computerized methods against manual methods [50,51], along with the variability of studies [28].

With the emergence of machine learning (ML), researchers used a single-layer feed-forward network (SLFN) for IMT boundary estimation [52,53]. While it uses knowledge of the database, it is also not fully automated as it involves some human interventions, particularly for grayscale feature extraction. This has been noticed well during the image-based tissue characterization in many fields of view [54,55,56,57,58]. Under the class of AI, deep learning (DL)-based methods have emerged over the past decade [59,60], offering the advantage of automated feature extraction. The DL-based methods use cascaded and sometimes parallel convolutional layers for feature extraction that can extract the fine details of the objects, which is sometimes not possible through ML-based feature extraction [26,52,61,62,63].

Our group has published nearly 100 machines learning journal publications and we strongly feel that while ML-based methods can be developed in a fully automated way, they, in general, require a more exhaustive method for optimization, unlike DL methods. Since the number of layers in ML-based methods are limited, the refining of features is not possible, unlike in the case of DL-based solutions. Our group has published over 50 deep learning journal publications and our observations prove that DL-based methods offer greater advantages compared to the ML-based methods.

Technically, DL-based methods fall into two categories: supervised and unsupervised. Supervised learning models learn from the labels (pixel or categorical labels or gold standard) provided along with the database, while unsupervised learning-based models do not use such labelled information. Cardiovascular imaging has widely used supervised learning-based methods. Cuadrado-Godia et al. [64] presented a two-stage model for cIMT and plaque measurement. Zhou et al. presented UNet and Unet++ models for plaque segmentation on multi-institutional databases [65,66]. Meshram et al. [67] applied the dilation factor on convolutional layers of the Unet model to study the effect on plaque segmentation.

Recently, a few studies have emerged regarding hybrid deep learning (HDL) models [68,69,70,71,72]. There is no specific definition or criteria for a hybrid deep learning model. Still, any modification in the basic encoder–decoder arms or skip connections of the fundamental UNet architecture may fall under the HDL category. In keeping the spirit of HDL, Jain et al. [73] were the first to propose a few hybrid deep learning (HDL) models for ICA plaque segmentation. The authors showed that HDL models are a fusion of two solo deep learning models with the feature extraction capability of two SDL models. Some faster, small, and low parameter HDL models were proposed by them recently [74]. Another study by Jain et al. [73] used the same HDL model on CCA plaque segmentation. Thus, HDL models are gaining more importance due to their better feature extraction capability.

**Figure 1:**
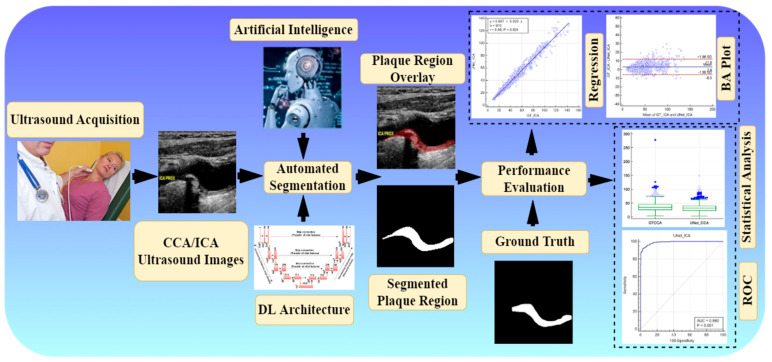
Global system (under the class of AtheroEdge™ 3.0) of carotid plaque segmentation.

Attention channel maps were recently introduced in UNet-based deep learning [75,76]; however, they have not been tested in the carotid ultrasound framework. Further, no study has yet benchmarked UNet against other models for a carotid ultrasound framework. Therefore, we hypothesize that attention channel maps, when added to the skip connection of UNet architectures, will improve the performance visually and quantitatively. Figure 1 shows the global system diagram of plaque segmentation using the attention-based UNet-based DL paradigm (named as AtheroEdge™ 3.0, AtheroPoint LLP, Roseville, CA, USA).

The layout of this study is as follows: Section 2 presents database selection, preparation, and baseline characteristics. Section 3 presents the architecture of All UNet models used in this study. Methodology and experiments are presented in Section 4. The results are presented in Section 5. Section 6 shows the performance evaluation, Section 7 presents the discussions, and the paper concludes in Section 8.

## 2. Database Selection, Preparation, and Baseline Characteristics

We considered multi-institution, multi-ethnic databases for our study. Hence, our experiments are free from data selection bias. We considered three databases in this research work, DB1: ICA database from the United Kingdom; DB2: CCA database from Japan; DB3: CCA database from Hong Kong. Detailed descriptions of the databases are provided here.

### 2.1. DB1: UK ICA Database

A video database of 99 B-mode ultrasound images was acquired from Imperial College London, UK. The database consisted of datasets from 47 male and 52 female patients with a mean age of 75.04 ± 9.96 years. Each US video was converted into still images. From each patient image pool, we selected 10 images at the interval of 10. After a retrospective analysis of US video scans, we found that two scans were fuzzy and noisy; therefore, we removed these two scans from the database. Finally, we achieved 970 images of moderate-to-high risk plaque images.

### 2.2. DB2: Japanese Diabetic CCA Database

DB2 was collected from Toho University, Japan. It consisted of 379 CCA images of 190 diabetic patients with a mean age of 68.78 ± 10.88 years. The database consisted of 147 male and 43 female patients. An experienced sonographer performed all the scans. This was a retrospective study, and the institutional review board approved the ethics. A detailed baseline characteristic was recorded at the time of diagnosis and presented in Table 1 based on the measured plaque area.

### 2.3. DB3: Hong Kong Post-Menopausal Women CCA Database

We collected the DB3 database from 50 Chinese women aged between 54 and 67 years. Further, we ordered six images from each patient; thus, the database constituted 300 images. The patients provided their written consent before the start of the experiment. All patients were post-menopausal women, and 28 such women were also identified and were suffering from other diseases. Of these 28 diseased women, one diabetic, three hypertensives, seven hypercholesteremic, fifteen hypertensive and hypercholesteremic, and two with all abnormalities were identified. The remaining patients had normal BP, cholesterol, and blood glucose levels in a fasting state.

### 2.4. Data Preparation and Augmentation Technique

Data preparation and augmentation play a vital role in DL-based systems. First, we removed the non-relevant information in ultrasound scans, such as patient ID (name), age, date, and model of the machine, etc., by cropping the grayscale area only. This is very popular in image processing and is a standardized method [45,77]. Secondly, we converted all images of DB1, DB2, and DB3 into equal-sized images of size 224 × 224, compatible with the first layer of the DL models. DB1 and DB2 are from different institutions and countries. Thus, we merged both databases to provide our experiments a multi-ethnic, multi-institutional database. Therefore, we combined the CCA images of DB2 and DB3 into a single folder, DB23, and achieved a new DB23 totaling 379 + 300 = 679 images. We also used a data augmentation technique on our combined CCA DB23 to enhance the number of images in the experiments. For this purpose, we used a rotation transform [–15° to +15°] on all images of combined CCA DB23. Finally, we accomplished a database DB2A, where A stands for augmentation of 679 × 2 = 1358 images.

### 2.5. Binary Mask Preparation for Supervised Learning

This work falls under the supervised learning-based semantic segmentation of the atherosclerotic plaque from carotid ultrasound images. Thus, the models require pixel-label information for the training phase. We have a team of experienced sonographers and cardiologists who successfully identified the LI and MA layers of the carotid artery and delineated the plaque. Further, another sonographer verified the same for any error. The delineated LI and MA closed borders (contours) in grayscale images are converted into a binary mask using a MATLAB-based program. Further, these binary masks are also converted into equal-sized images of 224 × 224, as with the greyscale image.

Inter- and intra-observer analysis always require a radiologist and sometimes become expensive. Further, while inter- and intra-observer studies were not an integral part of this pilot design, our observations has proven that such analysis leads to variations of between 1% and 5% [78,79,80,81,82,83,84,85,86,87,88]; such ranges are normal and meet the FDA 510 (K) regulations. We intend to integrate this practice in future studies

## 3. UNet Architectures

### 3.1. Basic UNet Model

**Figure 2:**
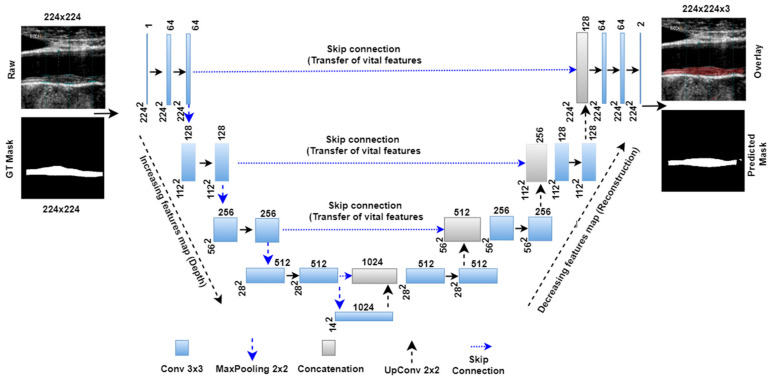
Basic Unet model with four encoder and decoder stages.

Figure 2 below shows the basic UNet model with four encoder and decoder stages and one bottleneck layer between them. The left part of the architecture forms the encoder arm of the network. Each encoder stage follows the convolutional, ReLU, convolutional, ReLU, and maxpooling layers. The numbers of convolutional filters double in each stage. Thus, the numbers of convolutional filters in successive stages are 64, 128, 256, and 512. The number of trainable parameters (P) after each stage was calculated using the following formula $P=\left[\left(m\times n\times d\right)\times b\times k\right]$, where *m* is the height of the convolutional filter, *n* is the width of the convolutional filter, *d* is the number of filters in the previous layer, *b* is the bias, and *k* is the number of filters in the current layer. The convolutional filter size is 3 × 3, and the default bias value (b) is 1 for each stage. Thus, after each encoder stage the number of the trainable parameters was 38,720, 221,440, 885,248, and 3,539,968, respectively. A bottleneck layer is present after the encoder stage, which carries the most delicate features extracted from the encoder stage. This bottleneck layer holds 14,157,824 numbers of trainable parameters. The right-hand part of the UNet architecture is called the decoder arm. The decoder stage follows the up-convolutional layer, followed by concatenation, convolutional, and ReLU layers.

During top to down parameters extraction in the encoder stage, vital semantic features are left in the extraction process. Thus, these features are added to the corresponding decoder stage at the concatenation layer via a skip connection (shown in the Figure 2 by dotted lines). After adding features from the encoder stage, each decoder stage carries 9,176,576, 2,294,528, 573,824, and 143,552 parameters. Finally, fully connected and softmax layers comprise 1154 and six parameters. Thus, Unet architecture contains a total of 31,032,840 training parameters. The Unet model is trained with grayscale images of 224 × 224 images and their corresponding binary masks. The model uses a sparse categorical cross-entropy loss function given by Equation (2) and an ADAM optimizer to reduce the loss function.

### 3.2. Unet++ Architecture

**Figure 3:**
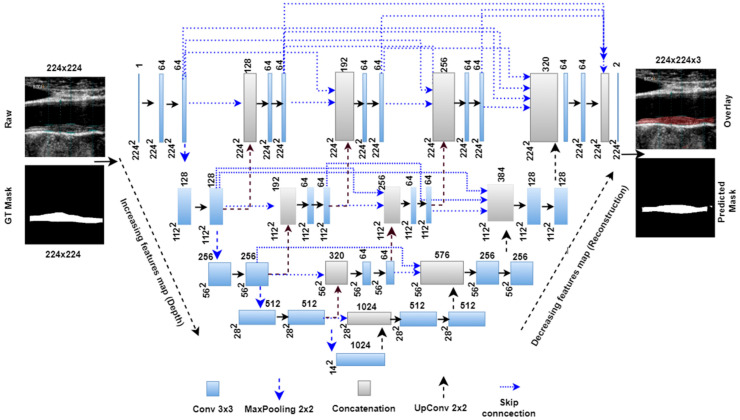
Unet++ model with four encoder and decoder stages.

The Unet++ network is a modified architecture of the basic Unet model. In the basic Unet model, the skip connection directly carries features from the encoder stage and concatenates to the decoder stage. However, the Unet++ model follows a dense skip connection path where features are passed through different intermediate blocks. As shown in the Unet++ architecture in Figure 3, the first, second, third, and fourth stage has 3, 2, 1, and 0 intermediate convolutional blocks. These intermediate blocks are connected to all previous blocks at the same level through the concatenation layer. The encoder arm has the same training parameters as the Unet model; however, in the decoder stage, training parameters change due to addition from the intermediate stages. Table 2 shows the number of training parameters in different parts of the Unet architectures.

### 3.3. Unet3P

**Figure 4:**
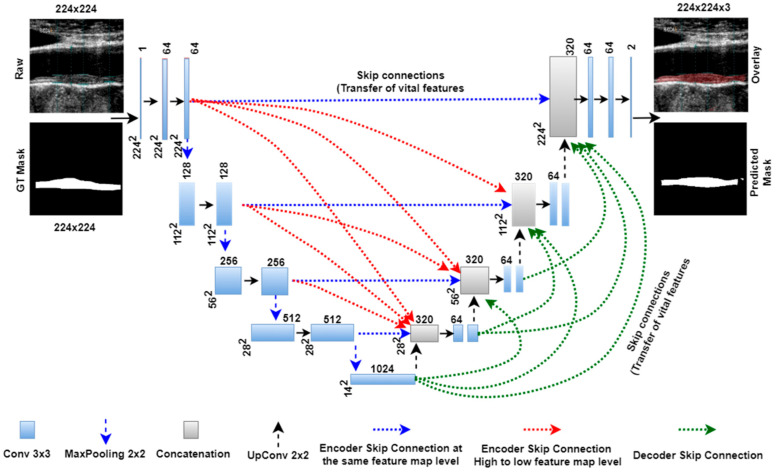
Unet3P model with four encoder and decoder stages.

Unet3P is another variant of basic Unet architecture. In the previous Unet and Unet++ models, the skip connections carry the features from the same encoder stage to the same decoder stage (same scale). However, multi-scale features exist at different encoder stages. Thus, these models lack in adding features from multi-scale feature connections. Therefore, the concept of adding multi-scale features to the Unet model arises as Unet+++ or Unet3P. This idiosyncratic Unet3P model merges features from the same-scale and lower-scale features from the encoder with the high-scale features from the decoder. As can be seen from Figure 4, the same-scale features from encoder stage 1, the large scale features from decoder stages 2, 3, and 4, and the bottleneck layers are added to decoder stage 1 via skip connections. Similarly, the lower-scale features from encoder stage 1, the same-scale features from encoder stage 2, and the large-scale features from decoder stages 3 and 4, as well as the bottleneck layers are concatenated to decoder stage 2 via skip connection. The lower scale features from encoder stage 1 and 2, the same-scale features from encoder stage 3, and the large-scale features from decoder stage 4 and the bottleneck layers are added to decoder stage 3. Finally, the lower-scale features from encoder stages 1, 2, and 3, the same-scale features from encoder stage 4, and the high-scale features from the bottleneck layer are concatenated with decoder stage 4. The complete architecture of Unet3P is shown in Figure 4.

### 3.4. Attention-based Unet Model

**Figure 5:**
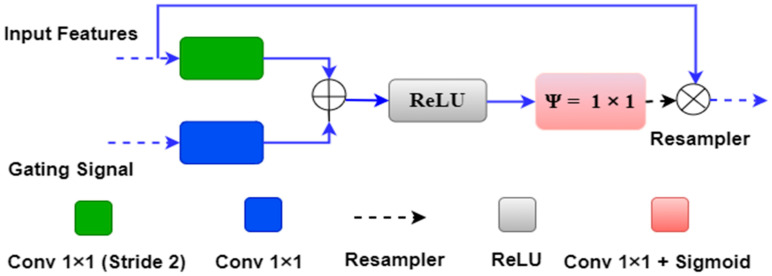
Attention block with input features, gating signals and weighted output.

The concept of the attention mechanism was proposed earlier by Bahdanau et al. [89] and Luong et al. [90]. Further, the same concept was integrated with the UNet by Oktay et al. [75] for segmentation of the Pancreas. We utilized the attention-UNet for plaque segmentation as the atherosclerotic plaque has a very fuzzy nature, which is challenging to segment using other UNet models in many cases. Figure 5 below shows an attention block used in the place of the skip connection of the UNet model.

The attention gate has two inputs and one output. One of the inputs is “input features” ‘x’ from the same encoder level, and the second input is gating features ‘g’ from the lower decoder level. Both inputs have an inherent property to carry the features. The input feature ‘x’ from the same encoder level comes from a shallow network level; therefore, it contains spatial feature information. The gating signal derives from the decoder level, which is at a deeper level compared to the input signal ‘x’. Thus gating signal provides better feature representation. Further, the input signal is downsampled by a stride of ‘2′ to make it compatible with the gating signal. The downsampled signal ‘x’ is then combined with the gating signal ‘g’ and passed through the rectified linear unit (ReLU) activation function. The ReLU is a non-linear activation function which removes the negative values from the input, i.e., it provides the output if it has positive values.

Further, both ‘x’ and ‘g’ signals are passed through a 1 × 1 convolutional operation to acquire the weights from the combined weight signal. The combined weight signal passes through a Sigmoid activation function. Sigmoid is a ‘S-shaped’ non-linear curve defined by Sig(x) = 1/(1 + exp(–x)). Due to its large slope value, it is able to transform the falling input values to between ‘0′ and ‘1′. Finally, the combined weight signal is upsampled to the same scale as the input signal ‘x’ and multiplied by it element-wise.

**Figure 6:**
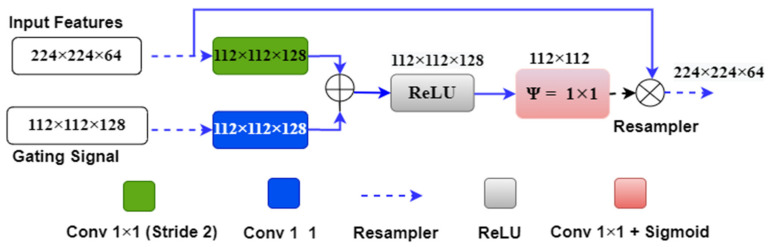
The attention gate mechanism between the first encoder and decoder layer.

We can understand the above attention mechanism with the example provided in Figure 6, which shows the first attention gate at the skip connection between the first encoder and decoder stages. The input features ‘x’ of size 224 × 224 × 64 originate from the first encoder stage, and the gating signal ‘g’ of size 112 × 112 × 128 comes from one lower decoder level. Input signal ‘x’ is downsampled by green block (convolutional 1 × 1 with #filter = gating signal filter i.e., 128) to the size 112 × 112 × 128. Also, the gating signal passes through the blue box (#filters = 128) with 1 × 1 convolution, so it acquires the same shape as ‘x’, i.e., 112 × 112 × 128. Both signals are added and applied to the ReLU activation function, where any nonlinearity is removed from the combined signal.

**Figure 7:**
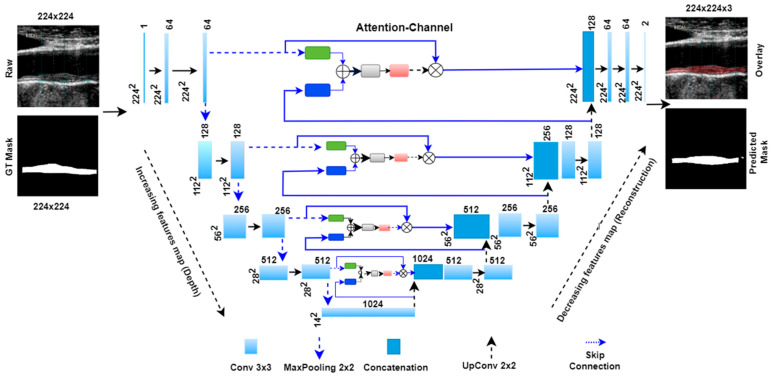
Four-stage Attention-UNet model.

Further, the combined signal is passed through the psi (Ψ) block, which is a 1 × 1 convolutional block, to acquire the weights from the combined signal. These weights are the attention gate weights generated by resampling the shallow and deep features from the encoder and decoder stages. These weights are upsampled to size 224 × 224 by the upsampler and multiplied to the input signal ‘x’ of the same size. Figure 7 shows the complete attention-UNet model with four encoder and decoder stages. In this architecture, four attention blocks are used in place of the skip connection between the encoder and decoder stages.

Table 2 comprises the training parameters of different parts of all UNet architectures, such as the encoder arm, decoder arm, bottleneck layer, intermediate stages, fully connected layer (FC), and classification layers. We can also compare the total number of parameters of individual models.

## 4. Methodology and Experiments

All six models were trained using the raw images and binary masks of the ICA DB1 and CCA DB2A mentioned in database Section 2. Various experimental setup steps and key points are mentioned below.

### 4.1. Hyperparameter Selection and Optimization

The choice of hyperparameters is highly standardized and well-established. The main parameters were (1) # of layers, (2) CV protocol, (3) # of epochs, (4) learning rate, (5) batch size, (6) filter size. The number of layers is shown in a block diagram of all DL models. Additionally, Table 2 shows the total number of parameters in major parts of the DL architecture. The hyperparameters of the experiments include 100 epochs, which we optimized after many experiments with the database. At this number of epochs, no significant change in loss function is observed, and the loss value converges. We selected batch sizes of 8 for UNet, UNet3P, Squeeze-UNet, and attention-UNet, and 4 for the UNet++ and Fractal-UNet models. Further, we used a standard learning rate of 10^−4^, convolutional filter size of 3 × 3, and bias value of 1 with the same padding.

### 4.2. Sparse Categorical Cross-Entropy Loss Function

We used a sparse-categorical cross-entropy loss function to minimize the training loss. The loss function is defined as the following Equation (1):

(1) $$L\left(w\right)=-\frac{1}{N}\;\sum_{i=1}^{N}\left[y_{i}\log\left(\hat{y_{i}}\right)+\left(1-y_{i}\right)\log\left(1-\hat{y_{i}}\right)\right]$$

where, *w* refers to the model parameters, e.g., weights of the neural network; N is the total number of pixels in an image, $y_{i}$ is the true (actual) label; and $\hat{y_{i}}$ is the predicted label.

### 4.3. K5 Cross-Validation

We implemented a commonly used K5 cross-validation [91] method for training and testing. The complete database is divided into 80% training and 20% test datasets in this cross-validation system. In the first step, the training set is used to train the system and offline weight generation, and the test set is used to validate the system and segmentation parameters generation. Again, we transferred the 20% test set back into the main dataset and used another 20% of the images for testing and 80% for training. We repeated the previous step. We repeated this training and testing experiment five times; thus, five offline models were generated corresponding to each test dataset. Now, each test dataset is used with a corresponding offline weight to generate the test results. Thus, all images are tested at least once.

Since the datasets were relatively small and our past experiences in AI protocols showed strong validation results [92], this pilot study did not conduct the validation component. This method can potentially be applied in subsequent experiments.

Experiment 1 is conducted with 970 ICA images. All images are divided into 776 training and 194 test images. The test set of 194 images is replaced with a unique set of 194 images from the training set. Finally, we test our system with five unique sets of the 194 test images. Similarly, experiment 2 is performed with 2 × 679 = 1358 images (DB2A) of Japanese and Hong Kong patients. The complete dataset is divided into five unique test datasets of 271, 271, 272, 272, and 272 images and their corresponding training sets. Each test set is used with the offline model generated after training on the corresponding dataset. Once the test result of each image is generated, we obtain the arithmetic mean of all images using the following Equation (2).

(2) $$X=\frac{1}{N}\sum_{i=1}^{N}x_{i}$$

where xi is the extracted feature of image ‘i’, and ‘X’ is the arithmetic mean of ‘N’ images.

## 5. Results

The segmentation performance of the six models with the ICA DB1 and CCA DB2A databases are shown in Table 3 and Table 4, respectively. We have acquired accuracy, sensitivity, specificity, precision, Mathew’s correlation coefficient (MCC), dice-similarity coefficient (DSC) and Jaccard Index (JI). These indices are calculated by comparing estimated binary masks generated from all UNet models and ground truth masks. The attention-based UNet show mean ± SD values of all parameters as 98.58 ± 0.59, 86.86 ± 5.73, 99.52 ± 0.38, 93.54 ± 4.62, 89.31 ± 3.76, 89.90 ± 3.69, and 81.86 ± 5.90 (all in %) respectively for ICA DB1 database. The same parameters acquire mean ± SD values of 99.01 ± 0.42, 81.75 ± 7.94, 99.74 ± 0.26, 92.71 ± 7.20, 86.38 ± 5.70, 86.50 ± 5.94, and 76.65 ± 8.36 (all in %), respectively, for the CCA DB2A database.

**Figure 8:**
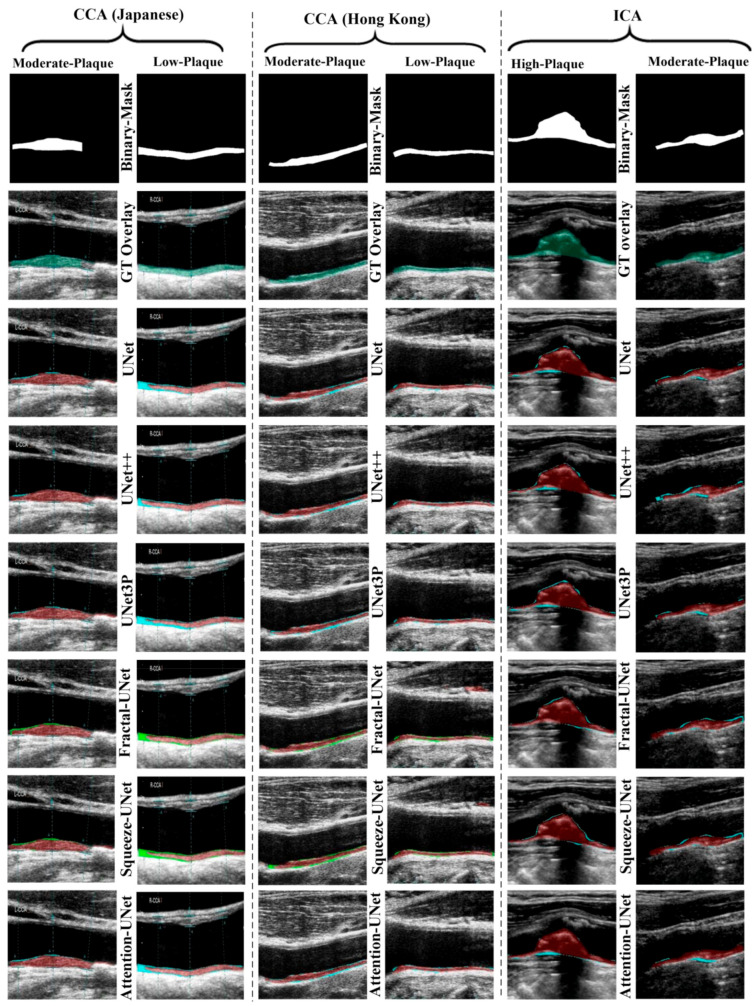
Visual results of the segmentation of Japanese, Hong Kong and UK (ICA) databases, performed by UNet, UNet++, UNet3P, and Attention-UNet models.

The visual results of UNet, UNet++, UNet3P, Fractal-UNet, Squeeze-UNet, and attention-UNet models are shown in Figure 8. The top row represents binary masks of all databases. The second-row shows overlays of the GT masks over the raw grayscale images in green colour. The third row shows the overlay of the difference between estimated and GT masks on raw grayscale images. Similarly, the fourth, fifth, sixth, seventh, and eighth rows also show the difference between the estimated and GT mask on raw grayscale images for UNet++, UNet3P, Fractal-UNet, Squeeze-UNet, and attention-UNet models, respectively. The red color indicates the estimated mask, and the green color represents the difference of the two masks.

**Figure 9:**
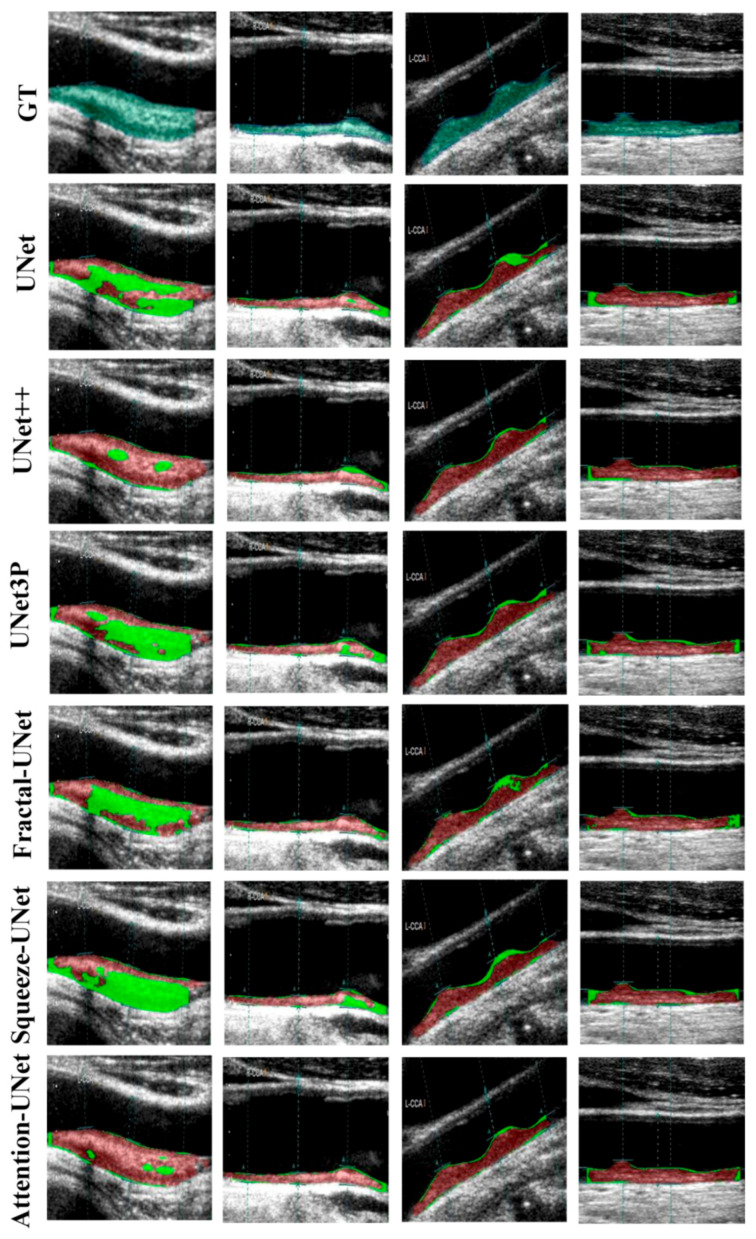
Attention channel effect on plaque segmentation on critical images of moderate and high plaque.

The attention mechanism states that the attention blocks modify the deep features by applying the attention weights. The same can be seen visually in some critical images shown in Figure 9, which are not successfully segmented using other models.

## 6. Performance Evaluation

The results of all models show almost equal segmentation indices. Hence we perform some more performance evaluation tests to validate our experiments. We conducted a series of performance tests on the ICA DB1 and DB2A databases, such as regression analysis, receiver operating characteristics (ROC) analysis, paired-*t*-test, and Bland–Altman’s plot analysis.

### 6.1. Regression Analysis

**Figure 10:**
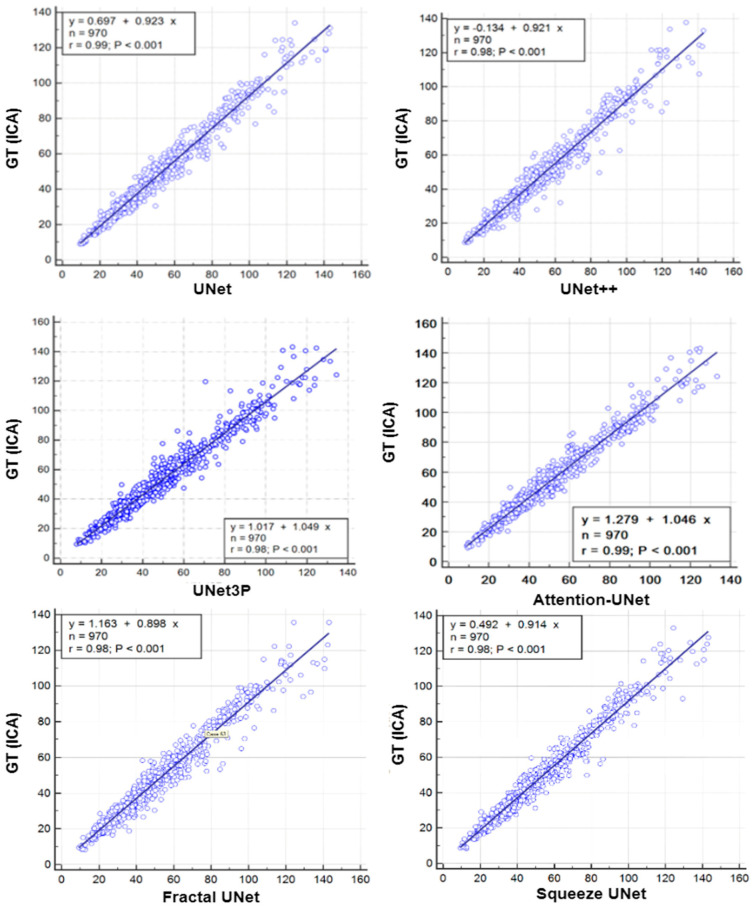
Regression plots for all 6 types of UNet models for ICA DB1 databases.

**Figure 11:**
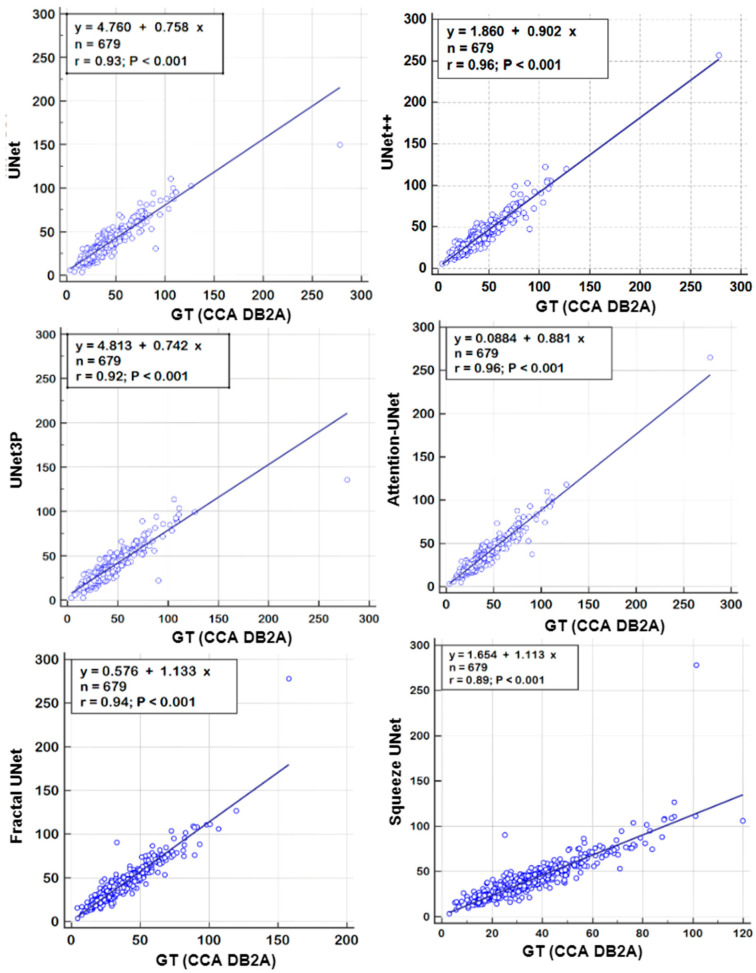
Regression Plot for all 6 types of UNet models for CCA DB2A database.

A regression analysis is a powerful statistical tool to analyze the relation between two quantities. It generates a correlation coefficient (CC) between the two variables, which occupies values between 0 and 1. A CC value close to ‘1’ refers to a very high correlation, whereas a CC close to ‘0’ refers to very little correlation between the two quantities. We used ground truth plaque area (GTPA) and predicted plaque area from the model for the regression analysis. Using ICA DB1, we obtained CC values between GTPA and PA from UNet, UNet++, UNet3P, Fractal-UNet, Squeeze-UNet and Attention-UNet of 0.99, 0.98, 0.98, 0.96, 0.96 and 0.99. Similarly, using CCA DB2A, the CC values for the same models are 0.93, 0.96, 0.92, 0.94, 0.89 and 0.96. All values are summarized in Table 5. The regression analysis curve is shown in Figure 10 and Figure 11. From the analysis of all CC numbers, it is clear that the attention-based UNet outperforms the other models.

### 6.2. Receiver Operating Characteristics

**Figure 12:**
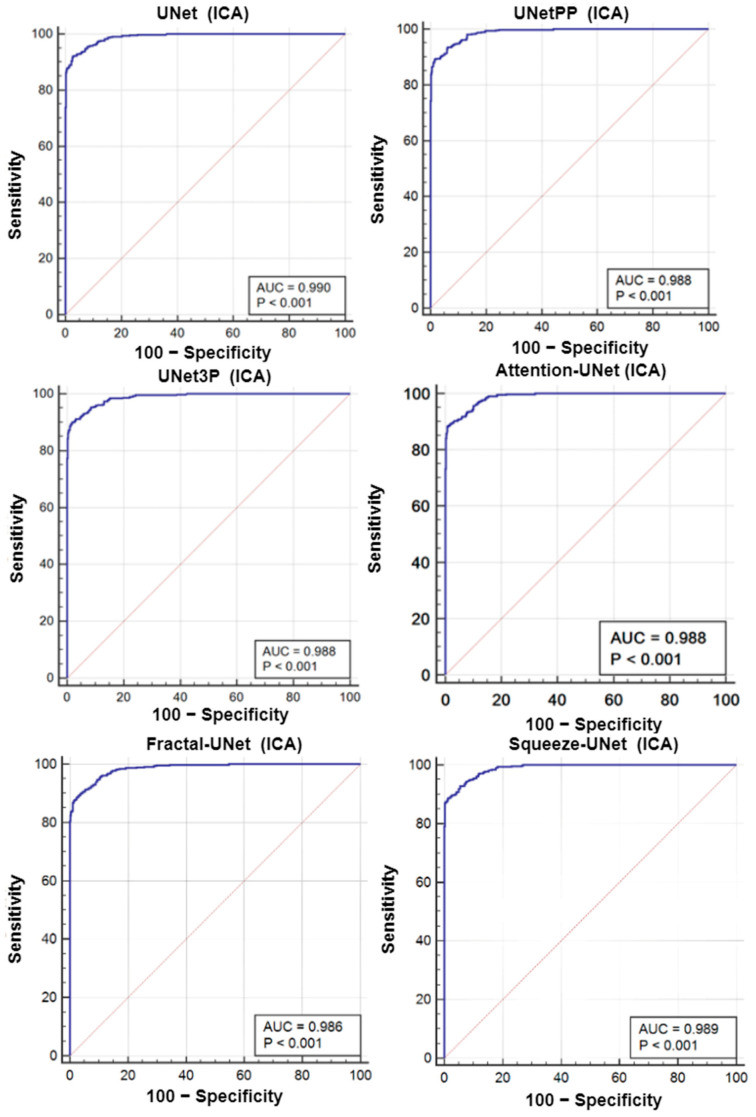
ROC curves for all 6 types of UNet model for ICA DB1 database.

**Figure 13:**
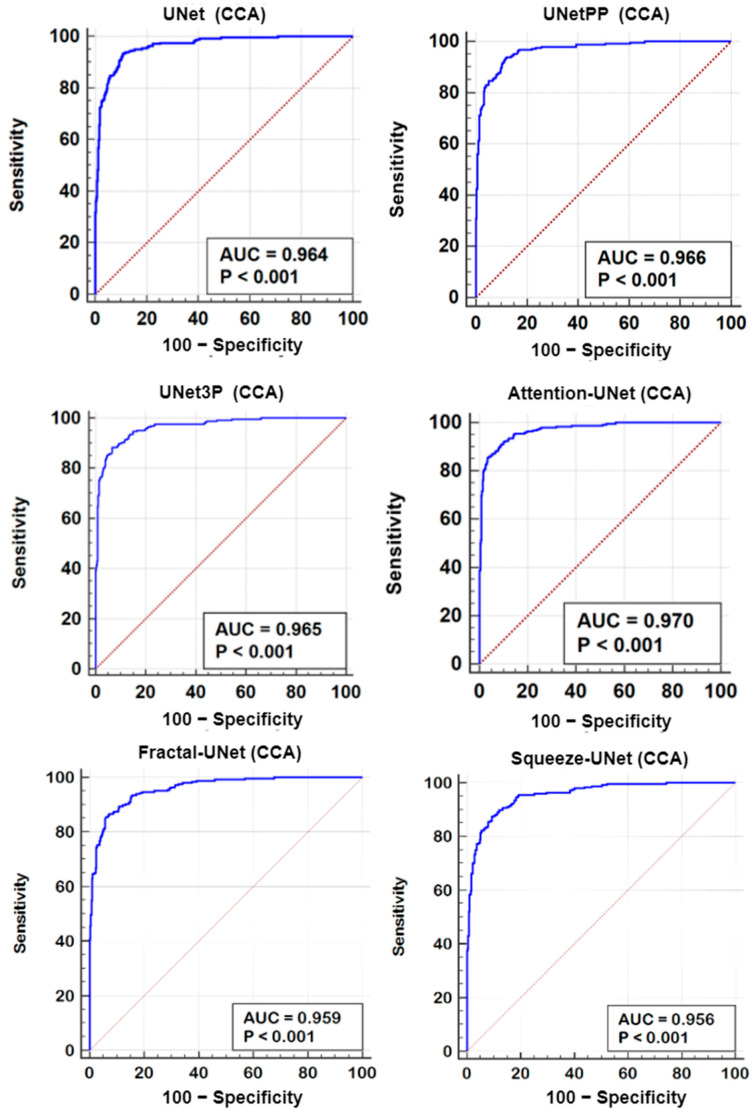
ROC Plot for all 6 types of UNet models for CCA DB2A database.

A receiver operating characteristics (ROC) analysis is another performance evaluation tool to assess the classification performance. We cross-examined the literature and found a plaque area threshold value of 40 mm^2^ used by researchers to classify the low and high risk plaque. Using this threshold value, we generated GT labels ‘1’ and ‘0’ for GTPA > 40 mm^2^ and GTPA < 40 mm^2^, respectively. Using the predicted area as the variable and GT labels as the classification variable, we plotted the ROC curve for UNet, UNet++, UNet3P, Fractal-UNet, Squeeze-UNet, and attention-based UNet models for ICA and CCA databases. These ROC curves are shown in Figure 12 and Figure 13 along with the area under the ROC curve (AUC) and *p*-values. The AUC values are compared in Table 5. Again, it is clear from the AUC numbers and the ROC curves that the attention-based UNet model outperforms other UNet models.

### 6.3. Paired-t-Test Analysis

**Figure 14:**
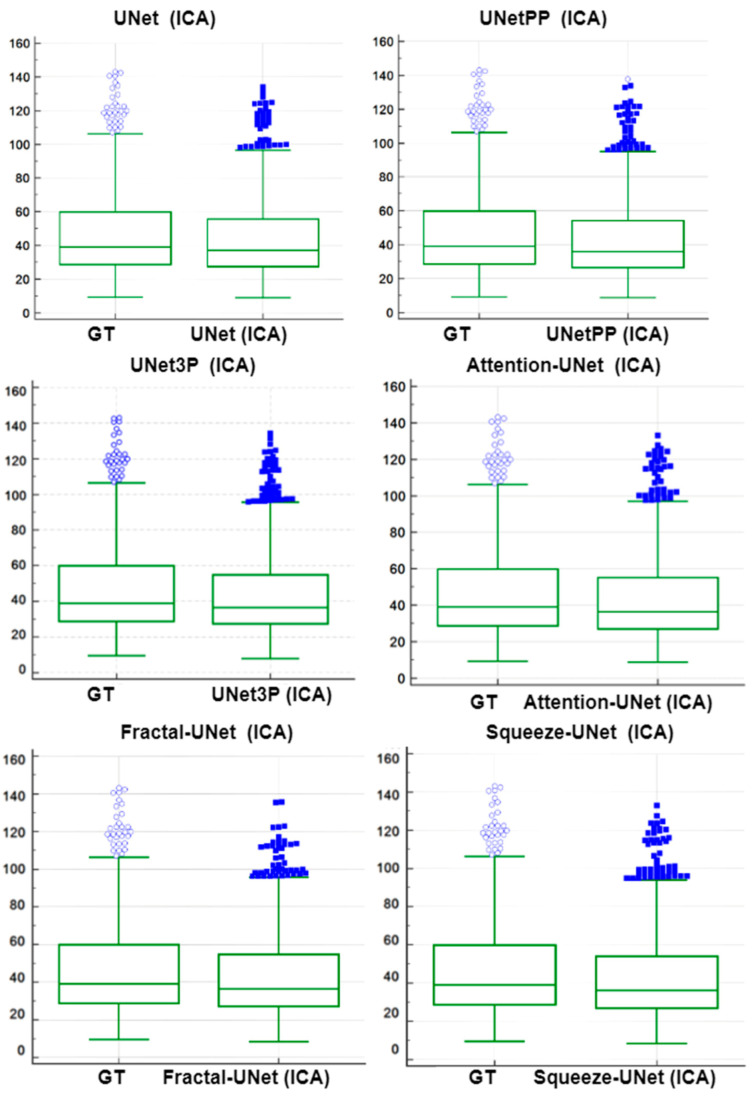
Paired samples *t*-test Plot for all 6 types of UNet models for ICA DB1 databases.

**Figure 15:**
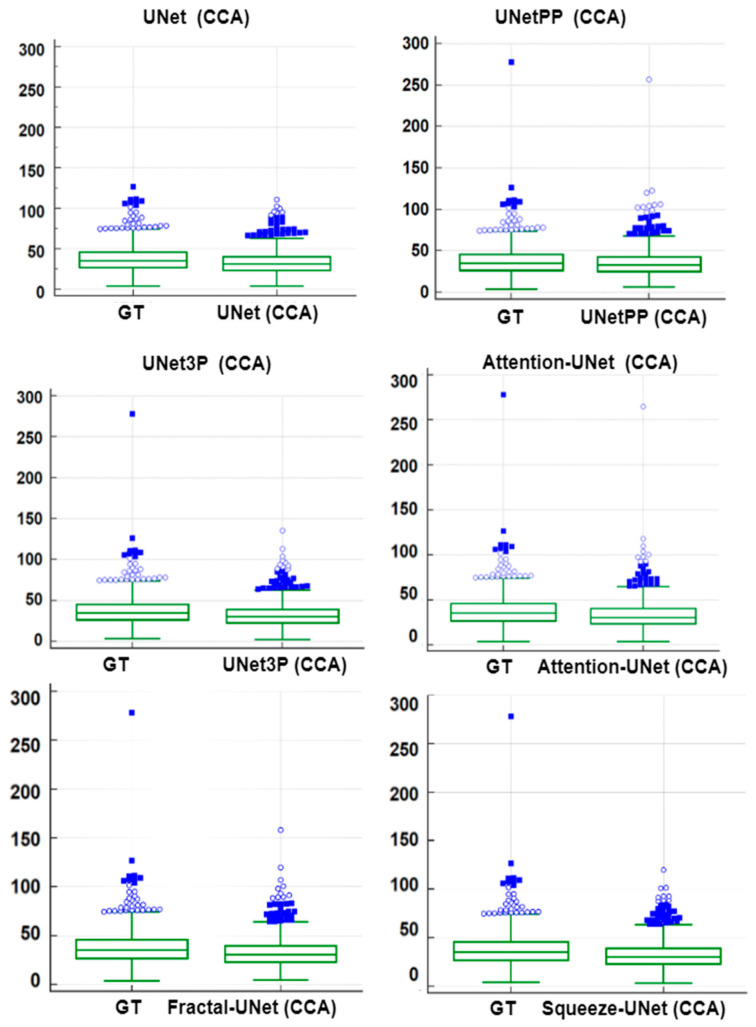
Paired samples *t*-test Plot for all 6 types of UNet models for CCA DB2A database.

A paired-*t*-test is mostly used in biostatistics to analyze the mean difference between the two measurements. The requirement for this test is the paired quantity of measurement for the same subject. In our case, we have GTPA and the predicted PA for the same arteries (CCA and ICA both). Thus, this method analyses whether the mean difference between the PA pair is zero or not. The distribution of GTPA and predicted PA is shown using the box and whiskers plot in Figure 14 and Figure 15 for ICA and CCA databases, respectively. The paired *t*-test results such as mean ± SD, the standard error of the mean, mean difference, Student’s t-value, and *p*-values are shown in Table 6 and Table 7 for the ICA and CCA databases, respectively.

### 6.4. Bland-Altman’s Plot

**Figure 16:**
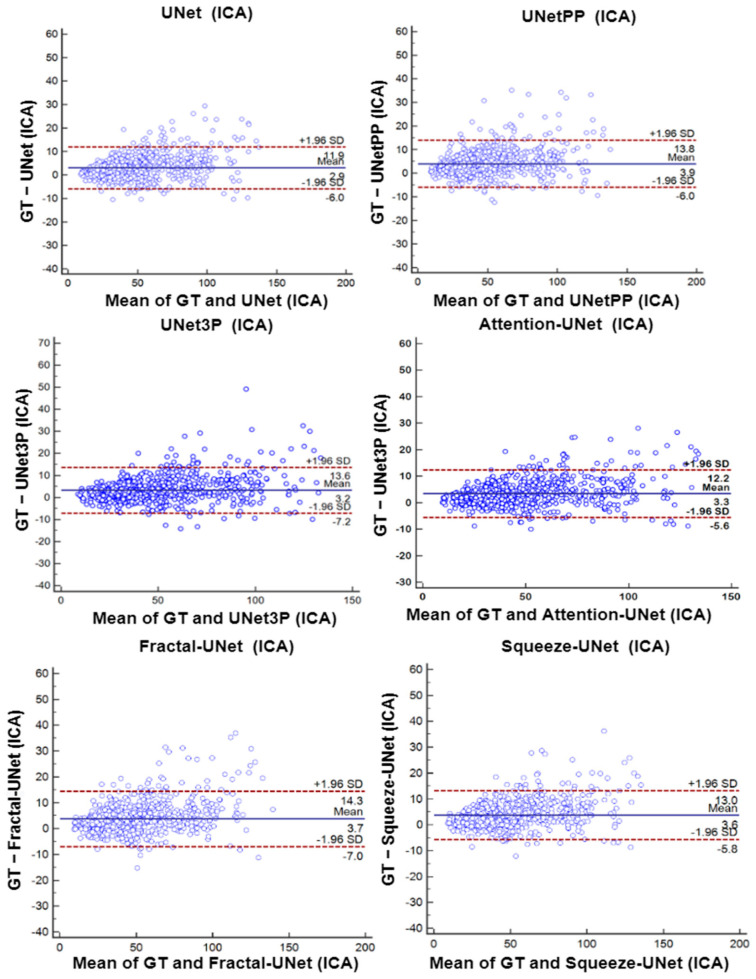
Bland–Altman Plot of all 6 types of models for ICA DB1 database.

**Figure 17:**
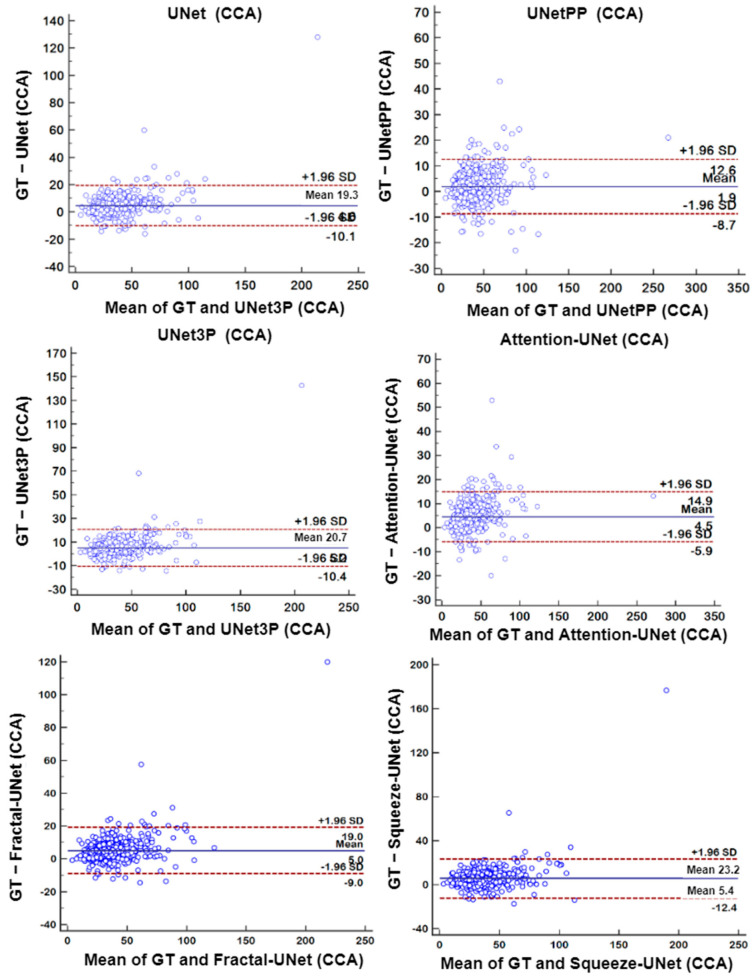
Bland–Altman Plot of all 6 types of models for CCA DB2A database.

A biostatistical analysis frequently uses a Bland–Altman plot or simply a difference plot when two different methods or instruments measure a parameter. The BA plot is used to show the bias between the mean differences of the two methods. It also offers an agreement interval of 95% (confidence interval), in which the difference between the second and first method fall. In our experiments, plaque area is the quantity which is measured by the expert sonographer by a manual method, i.e., GTPA and the predicted area measured by all UNet models. Thus, the difference between GTPA and the predicted PA is plotted on the Y-axis, and the mean of the both quantity is plotted on the X-axis. The BA plots between GTPA and predicted PA for all the UNet models using the ICA and CCA databases are shown in Figure 16 and Figure 17, respectively.

## 7. Discussion

The current research work is a novel application of attention-based UNet models for carotid plaque segmentation. We hypothesized that many of the images would be very fuzzy and bright to identify the plaque constituents. Thus, the attention-based UNet model [75,76] enhances the deep features and provides better segmentation. The model also shows excellent segmentation performance for low-to-moderate CCA and moderate-to-high ICA plaque images. Further, we compared the attention-based model against UNet, UNet++, and UNet3P models for a multicenter, multi-ethnic database. We made an effort to present a bias-free stroke risk assessment system.

### 7.1. Bias in Medical Imaging Models

Deep learning-based clinical models have recently been gaining significant attention. The models use clinical data available from hospitals and medical research centers. Further, these clinical data are processed using established algorithms or new algorithms. These AI-based clinical decision support systems have shown promising results in diagnosis. However, the biases in these systems are not reported in many studies [92,93]. These models may suffer from data selection bias (from single source); observer bias; data-labelling bias; data source bias (data device selection bias); validation bias; racial bias (multi-ethnic data selection); measurement bias; bias due to variabilities in the data sets [94], and many other types of bias which may affect the clinical results in some way. These biases must be discussed in detail and considered while designing the clinical support systems. Recently, methods have been developed to compute biases, which can be extended for UNet-based systems [95,96,97].

### 7.2. Supervised and Unsupervised Learning Based DL Models

The current algorithm uses binary masks of ICA and CCA images for model training. To successfully train the model’s error-free mask, preparation is a must; failure to prepare may result in the false recognition of the model. Binary mask preparation is tedious and time-consuming, and expert sonographers are required to accomplish this task. The current study involves the hundreds of images due to the unavailability of the datasets. However, considering the case where multiple thousands of images are available for analysis, generating such masks is nearly impossible. Therefore, supervised learning loses its significance in big data analysis. Thus, unsupervised models may replace such scenarios where no binary mask or labelled information is required for training. However, such unsupervised DL models have not gained much attention in medical image processing until now, and significant scope is available in this area. We may see many Unsup-DL models in the near future.

### 7.3. Benchmarking

We have presented an attention-channel-based UNet model for atherosclerotic plaque segmentation. Many efforts have been made in ICA and CCA plaque area segmentation; still, these methods are not perfect in one way or another. Previous methods suffer from some of the biases discussed in the above section, resulting in poor image segmentation from other databases. Zhou et al. [66] presented the UNet++ model for ICA and CCA plaque segmentation from multi-ethnic databases. However, they trained their model with only 33, 33, and 34 images and tested on 44 images. Using such a low number of images for the test does not infer proper justification to the clinical setting. Further, they did not benchmark their system with any other established system. In another study, Jain et al. presented hybrid deep learning models for ICA plaque segmentation [68]. They proposed SegNet-UNet and SegNet-UNet+ HDL models for plaque area segmentation. Although their model used only one segment of the artery, i.e., ICA, they enhanced the image database by applying the rotation transform augmentation technique. Thus, their models also suffer some biases such as data selection bias, source bias, and racial bias. However, their models are a milestone in HDL model studies.

In another study, the same team of researchers, Jain et al., used the above HDL models for plaque segmentation from multi-ethnic CCA databases [78]. They used two kinds of databases for the experiments, one from Japan and another from Hong Kong, and performed some unseen experiments. Thus, they attempted to avoid data selection and racial bias using these HDL models for unseen experiments. However, they failed to validate their experiments against any established system; therefore, their models suffer from validation bias. Further, they attempted to avoid the validation bias by comparing their SDL and HDL models against a commercially available state-of-the-art plaque segmentation system, AtheroEdge 2.0, developed by AtheroPoint LLC, CA, USA [73]. Their HDL model shows a plaque area error of 8 mm^2^ compared to 9.9 mm^2^ for SDL and 9.6 mm^2^ for AtheroEdge 2.0 models for 90% of the image database. The proposed method attempted to overcome previous bias by intensive exercises. By using databases from ICA and CCA sections, we attempted to avoid data selection bias. We also managed racial biases by incorporating multi-ethnic, multi-center ICA and CCA databases. DB1, DB2, and DB3 are from the UK, Japan and Hong Kong, respectively. Further, we validated our Attention-UNet model against previous UNet, UNet++, and UNet3P models to avoid model selection and validation bias. Table 8 summarizes the comparisons of the present study with some benchmark studies.

### 7.4. Strength, Weakness, and Extension

The present work shows a bias-free study of the plaque segmentation from ICA and CCA images from multi-ethnic, multicenter databases. We presented a powerful attention mechanism to modify the shallow and deep features of the carotid plaque images, which can capture those plaque areas which other models do not detect. We compared attention-based UNet segmentation results with other models used in previous studies, such as UNet, UNet++, and UNet3P, and the results are comparable or superior to such models. Moreover, the visual results show a promising improvement in many images. Further, we validated our experiments using a series of performance evaluation tests such as regression analysis, ROC, Bland–Altman plots, and paired-*t*-tests. The results of such performance tests are also comparable or superior to other models.

Further, we attempted to fill the gap in data selection and racial biases from previous studies using multicenter, multi-ethnic, and augmented databases. We believe that these biases are not sufficient to overcome, and there is still scope for their improvement, which can be attempted in future studies, as attempted here [98]. Further, the attention mechanism can be employed in other variants of the UNet to view its effect on other HDLs and such integration of advanced image processing [99] methods with UNet.

Since training models are large, one can adapt weight pruning techniques using algorithms such as genetic algorithms and whale optimization [100,101]. Moreover, the UNet-based segmentation method can be used for plaque tissue characterization due to its vital feature extraction and modification capability [71,102,103]. Carotid segmented lesions and plaque need to be correlated to a coronary SYNTAX score as part of clinical validation [104]. Segmented plaque can be attempted with different clinical groups such as rheumatology patients to understand cardiovascular risk [105]. Finally, since coronary plaque has been observed in COVID-19 patients [106], one can extend the UNet-based solution for plaque segmentation and measurement in carotid scans on COVID-19 patients.

## 8. Conclusions

This work presents a novel concept of the attention mechanism incorporated with UNet as an attention-based UNet model. The attention-based UNet model successfully demonstrated plaque segmentation in complex images with fuzzy and bright plaque. The results of the attention-UNet models were benchmarked against UNet, UNet++, and UNet3P models. The CC value of the attention-based UNet model for the CCA database was 0.96, compared to 0.93, 0.96, and 0.92 for UNet, UNet++, and UNet3P. The AUC value for attention-based UNet was 0.97, compared to 0.964, 0.966, and 0.965 for the other models. The attention gate weight modifies the shallow and deep features to identify the complex plaque images; therefore, the attention mechanism is vital in plaque feature extraction and tissue characterization. The system can be adopted in clinical settings for cardiovascular disease risk stratification.

## Figures and Tables

**Table 1:** Baseline characteristics of CCA cohort.

Risk Type	-		High-Risk		Low-Risk		-
PA Threshold	-		GTPA ≥ 40 mm^2^		GTPA < 40 mm^2^		-
N	Total (379)		48		331		-
Gender	293(M)	86(F)	37(M)	11(F)	256(M)	75(F)	
-	Mean	SD	Mean	SD	Mean	SD	*p*-values
Age	68.78	10.88	69.69	10.71	68.64	10.91	0.54
SBP	134.62	8.88	137.1	7.133	134.3	9.061	0.04
DBP	87.31	4.44	88.54	3.567	87.13	4.531	0.04
HbA1c	6.25	1.06	6.442	1.132	6.221	1.052	0.18
eGFR	45.67	20.43	43.77	23.35	45.95	19.99	0.49
LDL	100.84	31.48	101.6	29.01	100.7	31.87	0.87
HDL	50.60	14.73	47.63	12.63	51.03	14.98	0.14
TC	174.27	36.55	174.9	36.01	174.2	36.68	0.9
HT	277	-	41	-	236	-	<0.0001
Smoking	151	-	14	-	137	-	<0.0001
FH	47	-	7	-	40	-	<0.0001

**Table 2:** Number of trainable parameters in different parts of the UNet architectures.

Model	Encoder Arm	Bottleneck Layer	Decoder Arm	Intermediate Stages	FC	Classification Layer	Total Parameters
UNet	4685376	14157824	12188480	None	1154	6	31032840
UNet++	4685376	14157824	12593984	1275008	None	130	32712322
UNet3P	4685376	14157824	1196800	1447040	1154	6	21488200
Attention-UNet	4685376	14157824	12535680	523684	None	130	31902694

FC = fully connected layer.

**Table 3:** Segmentation performance (in %) of all UNet models using ICA database DB1.

Model	Acc	Sens	Spec	Prec	MCC	Dice	Jaccard
UNet	98.58 ± 0.61	87.43 ± 5.45	99.48 ± 0.40	93.11 ± 4.61	89.39 ± 3.63	90.02 ± 3.53	82.03 ± 5.66
UNet++	98.51 ± 0.65	85.72 ± 6.56	99.53 ± 0.38	93.70 ± 4.55	88.74 ± 4.14	89.32 ± 4.16	80.94 ± 6.45
UNet3P	98.51 ± 0.66	86.73 ± 6.35	99.47 ± 0.41	92.90 ± 4.84	88.87 ± 3.96	89.49 ± 3.90	81.19 ± 6.16
Fractal-UNet	98.41 ± 0.74	85.68 ± 6.71	99.45 ± 0.43	92.61 ± 5.35	88.13 ± 4.58	88.78 ± 4.50	80.10 ± 6.92
Squeeze-UNet	98.53 ± 0.63	86.29 ± 6.01	99.51 ± 0.39	93.40 ± 4.95	88.91 ± 4.09	89.52 ± 4.01	81.25 ± 6.30
Attention-UNet	98.58 ± 0.59	86.86 ± 5.73	99.52 ± 0.38	93.54 ± 4.62	89.31 ± 3.76	89.90 ± 3.69	81.86 ± 5.90

**Table 4:** Segmentation performance (in %) of all UNet models using CCA database DB2X.

Model	Acc	Sens	Spec	Prec	MCC	Dice	Jaccard
UNet	98.97 ± 0.53	81.58 ± 8.58	99.72 ± 0.27	92.20 ± 7.39	85.97 ± 5.77	86.07 ± 6.17	76.01 ± 8.59
UNet++	99.05 ± 0.38	85.82 ± 7.21	99.62 ± 0.31	90.04 ± 8.09	87.23 ± 5.43	87.48 ± 5.57	78.14 ± 8.01
UNet3P	98.93 ± 0.56	80.39 ± 9.09	99.73 ± 0.26	92.42 ± 7.25	85.43 ± 6.24	85.49 ± 6.68	75.18 ± 9.00
Fractal-UNet	98.91 ± 0.54	80.08 ± 8.67	99.72 ± 0.27	85.17 ± 6.56	91.96 ± 7.61	85.06 ± 6.29	74.68 ± 8.92
Squeeze-UNet	98.90 ± 0.63	79.67 ± 10.45	99.73 ± 0.27	84.94 ± 7.56	92.33 ± 7.09	84.94 ± 7.60	74.48 ± 9.98
Attention-UNet	99.01 ± 0.42	81.75 ± 7.94	99.74 ± 0.26	92.71 ± 7.20	86.38 ± 5.70	86.50 ± 5.94	76.65 ± 8.36

**Table 5:** Performance parameters of all UNet models for ICA and CCA database experiments.

	ICA			CCA		
Model	CC	AUC	*p*-Values	CC	AUC	*p*-Values
UNet	0.99	0.99	*p* < 0.001	0.93	0.964	*p* < 0.001
UNet++	0.98	0.988	*p* < 0.001	0.96	0.966	*p* < 0.001
UNet3P	0.98	0.988	*p* < 0.001	0.92	0.965	*p* < 0.001
Fractal-UNet	0.96	0.962	*p* < 0.001	0.94	0.959	*p* < 0.001
Squeeze-UNet	0.96	0.969	*p* < 0.001	0.89	0.956	*p* < 0.001
Attention-UNet	0.99	0.988	*p* < 0.001	0.96	0.97	*p* < 0.001

**Table 6:** Paired-*t*-test metrics for GTPA and estimated area by all UNet models for ICA database.

Models	Mean ± SD	Std Error of Mean	Mean Difference	SD of Differences	Std Error of Mean Difference	95% CI	Test Statistic-t	*p*-Value
UNet	44.5496 ± 24.2130	0.7774	−2.9449	4.5716	0.1468	−3.2330 to −2.6568	−20.063	<0.0001
UNet++	43.5908 ± 24.2386	0.7783	−3.9037	5.0739	0.1629	−4.2234 to −3.5840	−23.962	<0.0001
UNet3P	44.2960 ± 24.1313	0.7748	−3.1985	5.3020	0.1702	−3.5326 to −2.8644	−18.789	<0.0001
Fractal-UNet	43.8306 ± 23.6977	0.7609	−3.6639	5.4349	0.1745	−4.0063 to −3.3314	−20.996	<0.0001
Squeeze-UNet	43.8814 ± 23.9853	0.7701	−36131	4.7915	0.1538	−3.9150 to −3.3112	−23.485	<0.0001
Attention-UNet	44.1855 ± 24.3427	0.7816	−3.3090	4.5570	0.1463	−3.5961 to −3.0219	−22.615	<0.0001

GTPA: Mean = 47.4945 mm^2^; SD = 25.8415 mm^2^.

**Table 7:** Paired-*t*-test metrics for GTPA and estimated area by all UNet models for CCA database.

	Mean ± SD	Std Error of Mean	Mean Difference	SD of Differences	Std Error of Mean Difference	95% CI	Test Statistic-t	*p*-Value <
UNet	34.0097 ± 15.8775	0.6093	−4.5858	7.5137	0.2883	−5.1520 to −4.0197	−15.904	0.0001
UNet++	36.6595 ± 18.2781	0.7014	−1.9361	5.4177	0.2079	−2.3443 to −1.5278	−9.312	0.0001
UNet3P	33.4363 ± 15.6883	0.6031	−5.1592	7.9247	0.3041	−5.7564 to −4.5621	−16.964	0.0001
Fractal-UNet	33.5501 ± 16.1022	0.6179	−5.0454	7.1421	0.2741	−5.5836 to −4.5073	−18.408	0.0001
Squeeze-UNet	33.1997 ± 15.5589	0.5971	−5.3958	9.0953	0.349	−6.0811 to −4.7105	−15.459	0.0001
Attention-UNet	34.0731 ± 17.8005	0.6831	−4.5224	5.3039	0.2035	−4.9221 to −4.1228	−22.218	0.0001

GTPA: Mean = 38.5955 mm^2^ SD = 19.4775 mm^2^.

**Table 8:** Benchmarking current research against previous studies.

Authors	Artery Segment	DL Model	#Patients/#Images	Results	Bias Identified
Zhou et al. [66]	ICA, CCA	UNet++	N1 = 144/510 N2 = 497/638	TPA error: 5.55 ± 4.34 mm^2^	Data selection, model selection, validation bias
Jain et al. [68]	ICA	UNet, UNet+, SegNet, SegNet-UNet, SegNet-UNet+,	N = 97/970	PA error 3.49 mm^2^ for SDL; 4.21 mm^2^ for HDL	Data selection bias
Jain et al. [78]	CCA	UNet	N1 = 379 N2 = 300	FoM of 70.96 and 91.14 (unseen) against 97.57, 88.89, and 99.14 (seen)	Validation bias
Jain et al. [73]	CCA	UNet, SegNet-UNet, AtheroEdge 2.0	N1 = 379	PA error HDL = 8 mm^2^ SDL = 9.9 mm^2^ AtheroEdge 2.0 = 9.6 mm^2^	Data selection bias, racial bias
Proposed method	ICA, CCA	UNet, UNet++, UNet3P, Attention-UNet	N1 = 970; N2 = 379; N3 = 300	CC: 0.99 and 0.96 for ICA and CCA experiments	Free from data selection, racial, and validation biases

## Data Availability

The Institutional Review Board has been issued to AtheroPoint, Roseville, CA, USA and therefore this database cannot be shared publically.
